# Cyclooxygenase-2 immunohistochemical expression is associated with worse prognosis in breast cancer: Retrospective study and literature review

**DOI:** 10.15537/smj.2022.43.7.20220052

**Published:** 2022-07

**Authors:** Jaudah Al-Maghrabi, Mohamad N. Khabaz

**Affiliations:** *From the Department of Pathology (Al-Maghrabi), Faculty of Medicine; from the Department of Pathology (Khabaz), Rabigh Faculty of Medicine, King Abdulaziz University, and from the Department of Pathology (Al-Maghrabi), King Faisal Specialist Hospital and Research Centre, Jeddah, Kingdom of Saudi Arabia.*

**Keywords:** cyclooxygenase-2, COX-2, breast cancer, immunohistochemistry

## Abstract

**Objectives:**

To assess the immunohistochemistry phenotype of cyclooxygenase-2 (COX-2) in breast cancer (BC) and to correlate it with histological and clinical prognostic factors.

**Methods:**

This retrospective study utilized COX-2 monoclonal antibody in an immunohistochemistry staining of tissue microarrays slides of 570 cases of previously diagnosed BC and with 52 of normal breast tissues from breast specimens resected for benign lesions or reconstruction (fibroadenoma and normal breast epithelium). This project was carried out in the Laboratory of pathology, King Abdulaziz University, Jeddah, Saudi Arabia, between September 2019 and September 2021.

**Results:**

The present data showed an important connection between the COX-2 expression phenotype and BC compared to benign breast tissues (*p*=0.034). The expression pattern of COX-2 was allied significantly with some factors which distinguished aggressive subtypes of BC, such as stage, distant metastases, lymphovascular invasion, and poor survival.

**Conclusion:**

Cyclooxygenase-2 is a valuable marker that could facilitate BC diagnosis and prognosis.

**T**he cyclooxygenase enzyme (COX) family includes 3 isoforms.^1^ Cyclooxygenase-2 (COX-2) is an induced membrane bound isoform; its tissue expression is controlled by growth factors, endotoxins, and some cytokines (namely, interleukin 6, interleukin 1 beta, or tumor necrosis factor alpha), thus upregulated in inflammation. The encoding gene of COX-2 was found on chromosome 1.^2-3^ Cyclooxygenase-2 protein displays a considerable homology (60%) with COX-1; also, COX-2 exhibits a carboxyl-terminus extension and a diverse binding area for non-steroidal anti-inflammatory drugs, which presents COX-2 as a favoured aim in comparison with COX-1, consequently, will be repressed at smaller doses.^4,5^

Cyclooxygenase-2 is accountable for increased production of prostaglandin E2 that enhance the alteration of several procarcinogen effects.^6^ It is another molecular target that has been shown to have significance in cancer development. Oncogenic viruses, cancer promotors, radiation, and chemotherapy and proinflammatory cytokines are activators of COX-2 expression in transformed cells.^7-9^

Augmented COX-2 expression was defined in the pathogenic process of a broad selection of tumors and was found to induce activities like those of cancer stem cell and supports apoptosis resistance, proliferation, invasion, and metastasising of malignant cells.^3,10-12^ Cyclooxygenase-2 promotes carcinogenesis, raises the recurrence rate of cancer, and reduces survival in cancer patients.^13-15^ It also increases the resistance of malignant cell to radiotherapy and chemotherapy.^16^ Regarding COX-2 phenotype in breast cancer (BC), in the last decade, 17 studies examined the correlation among COX-2 and some of the histopathologic parameters of BC, the results were controversial and need further confirmation.^17-33^

Malignant neoplasms of breast are the most common malignancy in females around the world and is listed second as a cancer death cause after lung cancer. It has severe effects on women’s health worldwide.^34^ Information at the national level in Saudi Arabia showing the severity of BC requires more attention. As reported by the Cancer Registry of Saudi Arabia, breast neoplasms are the most frequent malignancies in Saudi females, and younger females are more and more affected by BC.^35^

This study aimed to study the immunohistochemical phenotype of COX-2 in BC and to correlate it with histological and clinical and prognostic factors.

## Methods

A retrospective study was carried out, between September 2019 and September 2021, and included a total of 570 BC specimens surgically removed prior to radio therapeutic, chemotherapeutic and hormonal manipulation regimes, which were investigated and examined by immunohistochemical staining, along with 52 of normal breast tissues from breast specimens resected for benign lesions or reconstruction (fibroadenoma and normal breast epithelium) were used as controls. The data of patients and histopathology blocks and slides were gathered from the Pathology Department, King Abdulaziz University Hospital, Jeddah, Saudi Arabia.

Tumor grade was reviewed and reclassified in line with the classification of the World Heatlh Organization (WHO).^36^ The tumor stage was reviewed and reclassified in line with the standards of the American Joint Committee on Cancer.^37^ Stage was categorized as low stage cancers (Stages 0-II) and high stage cancers (Stages III-IV). The clinical and pathological outcomes are recorded in Table 1. This study was permitted by the Biomedical Ethics Committee at King Abdulaziz University, Jeddah, Saudi Arabia. The applied practices and techniques were compliant with the revised Helsinki Declaration.

**Table 1:** - Clinicopathological parameters of tumors (N=570).

Parameters and total number of cases	n (%)	Coxa immunoexpression		*P*-values
		Negative	Positive	
***Type of tissue***				
Breast cancer	570 (100)	331 (57.3)	239 (42.7)	0.034
Normal breast	52 (100)	38 (73.1)	14 (26.9)	
***Gender***				
Male	5 (0.9)	2 (40.0)	3 (60.0)	0.351^*^
Female	565 (99.1)	329 (58.2)	236 (41.8)	
***Age***				
<50 years	312 (54.7)	186 (59.6)	126 (40.4)	0.240^*^
≥50 years	258 (45.3)	145 (56.2)	113 (43.8)	
***Grade***				
Grade 1	95 (16.7)	64 (67.4)	31 (32.6)	0.193^*^
Grade 2	285 (49.8)	160 (56.1)	125 (43.9)	
Grade 3	190 (33.5)	107 (56.3)	83 (43.7)	
Invasive ductal	489 (88.9)	290 (59.3)	199 (40.7)	0.130^*^
Others	71 (11.1)	36 (50.7)	35 (49.3)	
***Tumor size** (n=492)*				
<3	181 (36.8)	111 (61.3)	70 (38.7)	0.112
3-6	229 (46.5)	129 (56.3)	100 (43.7)	
>7	82 (16.7)	39 (47.6)	43 (52.4)	
***Pathological stage (n=495)***				
Low stage	333 (67.3)	207 (62.2)	126 (37.8)	0.001^*^
High stage	162 (32.7)	75 (46.3)	87 (53.7)	
***Nodal metastasis (n=478)***				
Negative	169 (35.4)	96 (56.8)	73 (43.2)	0.470^*^
Positive	309 (64.6)	173 (56.0)	136 (44.0)	
***Distant metastasis (n=450)***				
Negative	389 (86.4)	237 (69.9)	152 (39.1)	0.003^*^
Positive	61 (13.6)	25 (41.0)	36 (59.0)	
***Lymphovascular invasion (n=412)***				
Negative	225 (54.6)	138 (61.3)	87 (38.7)	0.034^*^
Positive	187 (45.4)	97 (51.9)	90 (48.1)	
***Surgical margins (n=445)***				
Negative	377 (84.7)	217 (57.6)	160 (42.4)	0.999^*^
Positive	68 (15.3)	39 (57.4)	29 (42.6)	
***Local disease recurrence (n=300)***				
Negative	220 (73.3)	132 (60.0)	88 (40.0)	0.072^*^
Positive	80 (26.7)	39 (48.7)	41 (51.3)	

Values are presented as numbers and percentage (%).*Chi-square test

The assembly of tissue microarrays (TMA) was carried out as explained in our previous published reports.^38,39^ Hematoxylin and eosin-stained slides of BC, fibroadenoma, and normal breast epithelium were assessed, and chosen areas were marked. Cases that exhibited widespread necrosis, poor cells’ preservation, inadequate tumor tissue, or cellular autolysis were excluded. Paraffin blocks of chosen cases were employed to obtain 2 cores of tumor tissue and next impeded in blocks by TMA Master 1.14 SP3 - a tissue microarray machine - (3DHISTECH Ltd., Budapest, Hungary). Then sections (4 μm) were sliced and used for immunohistochemistry staining technique.

Tissue microarrays blocks have been sliced at 4 μm and put on coated slides. Deparaffinization and rehydration of sections were completed using an auto-immunostainer (Ventana Medical Systems Inc., Tucson, USA). Immunohistochemistry stain was carried out utilising a diluted monoclonal antibody for COX-2 (1:50) (Dako, Glostrup, Denmark). Positive colorectal carcinomas for COX-2 have been employed as positive control. Breast cancer slides with replacement of the monoclonal antibody with Tris-buffered saline were utilized as a negative control.

The fraction of COX-2 positive cells was semi-quantitatively recorded. Slides with cytoplasmic COX-2 staining were described as positive. Staining intensity was scored strong (3), medium (2), weak (1), or absent (0). Cases with positive tumor cells of less than 5% were considered negative.

### Statistical analysis

All information was evaluated using the Statistcal Package for the Social Sciences, version 21.0 (IBM Corp., Armonk, NY, USA). All findings were recorded in numbers and percentages. Association between clinicopathological information of BC and COX-2 phenotype was examined via Chi-squared and Fisher tests. The Cox proportional hazards model helped decide if any of the clinicopathological factors have an important influence on overall survival (OS) and disease free survival (DFS). Evaluation of survival distributions for various COX-2 expression scores used Kaplan-Meier survival curve. *P*-values of <0.05 were counted statistically important.

## Results

**Figure 1:**
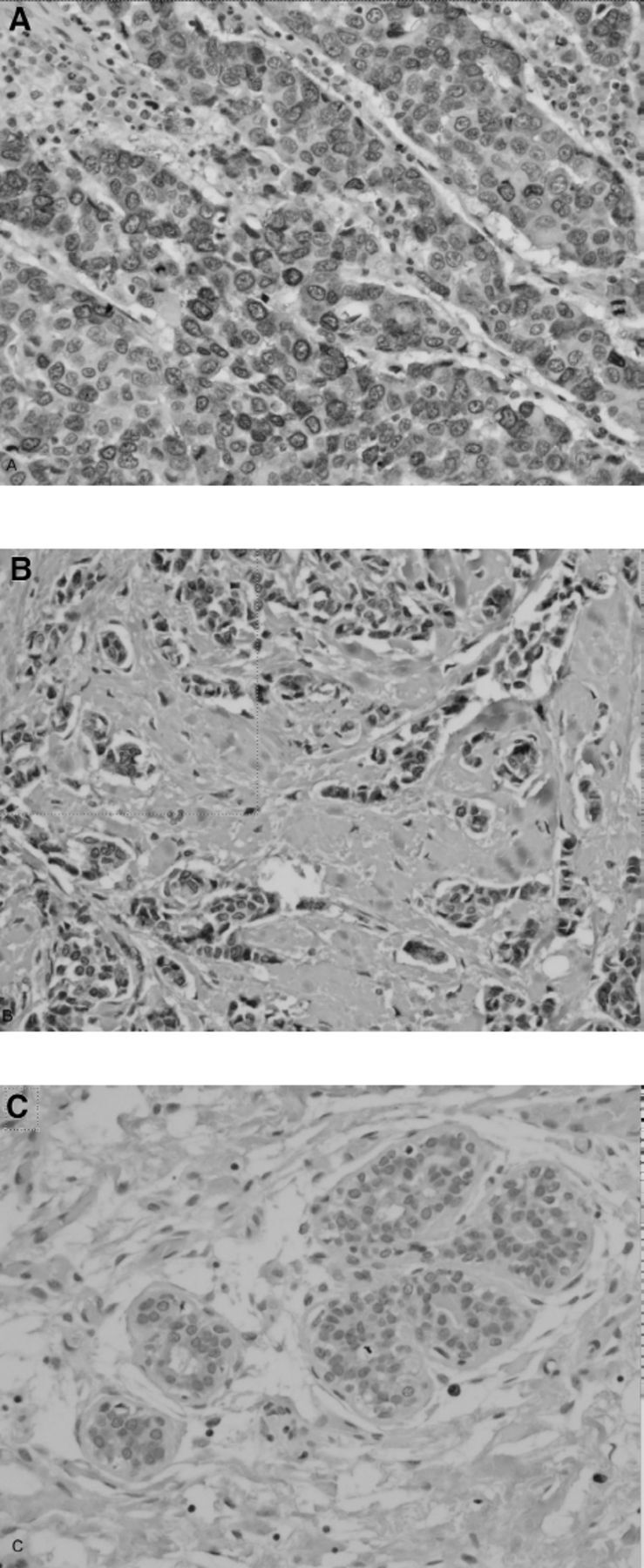
- Cyclooxygenase-2 (COX-2) immunohistochemistry staining patterns in breast cancer. A) Strong COX-2 staining in breast cancer (40 X). B) Moderate COX-2 staining in breast cancer (40 X). C) Weak COX-2 staining in breast cancer (40 X).

Cyclooxygenase-2 was expressed in the cytoplasmic part of malignant epithelial cells as brown granular staining in 239 (42.7%) cases of BC (Figure 1) and was detected in 14 (26.9%) of normal breast tissue (Table 1). The present data show an important association between COX-2 phenotype and BC compared to benign breast tissue (*p*=0.034). Fibroblasts and other interstitial cells were infrequently stained with COX-2.

Table 1 displays the status of COX-2 phenotype in BC cases and its correlation with several pathological and clinical factors. The percentage of positive malignant cells varies between 5-100% in BC of the current study. Almost half of BC cases revealed positive COX-2 staining in greater than 50% of their malignant epithelial cells. Stage of BC is correlated significantly with escalated COX-2 immunoexpression (*p*=0.001). A substantial portion of high-stage cases was observed to be frequent with positive COX-2 staining. Considerably, more tumors with distant metastases were detected with positive COX-2 staining (*p*=0.003). Almost half of the cases with lymphovascular invasion showed positive COX-2 staining (*p*=0.034). Local recurrence of the tumor was marginally correlated with positive COX-2 immunostaining cases (*p*=0.072). Recurrence is less common in negative COX-2 staining cases. No association was detected among COX-2 immunohistochemical phenotype and age, gender, size, lymph node metastasis, or margin status.

**Figure 2:**
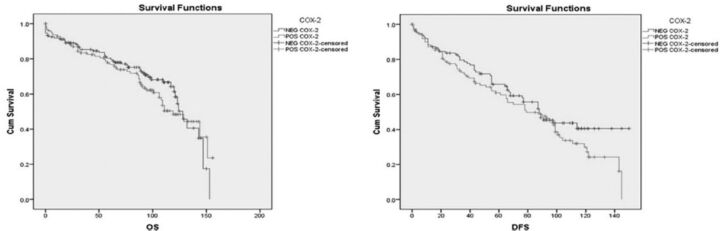
- Overall survival (OS) and disease-free survival (DFS) curves (Kaplan Meier) in relation to cyclooxygenase-2 (COX-2) immunoexpression in breast cancer patients (There is an association between COX-2 immunostaining and OS [log-rank: 4.136, *p*=0.042], and DFS [log-rank: 5.968, *p*=0.015]).

The results of Log Rank test showed that substantial diverse survival distributions are found for various scores of COX-2 staining. The statistics reveals that COX-2 immunoexpression is correlated with the probabilities of DFS (log rank: 5.968, *p*=0.015) and OS (log rank: 4.136, *p*=0.042) (Figure 2). Positive COX-2 immunostaining is related to poor survival significantly.

## Discussion

Although the immunoexpression of COX-2 has been widely studied in BC tissues (Table 2). Over the past decade, 17 reports employed immunohistochemistry staining to describe the phenotype of COX-2 in BC and to associate this expression with the clinicopathological parameters of BC cases, but the results showed a considerable controversy. Four of these studies showed statistically significant overexpression of COX-2 in BC tissues compared to that in benign tissues of breast, and potential clinical use of COX-2 in prognosis prediction.^17-20^ These results are consistent with our report that addressed a significant increase (*p*=0.034). While only 2 studies found opposite results and stated that COX-2 expression is more common in normal and benign lesions of the breast compared with BC, and the remaining studies did not investigate COX-2 immunoexpression in normal tissue and benign lesions of the breast.^21,22^

**Table 2:** - Studies of cyclooxygenase-2 expression in breast cancer.

References	Normal breast tissues	BC tissues	Age	Tumor size	Histologic type	Grade	Stage	Lymph nodes metastasis	Distant metastasis	Lymphovascular invasion	Surgical margins	Disease recurrence	Survival
Present study	26.9%	42.7%	-	-	-	-	+	-	+	+	-	-	+
Chen et al^17^	16.7%	72.7%	-	-		+		+	+				+
Wang et al^18^	15.4%	78.9%	-	-		+		+	+	+			+
Muhammad et al^19^	44%	72.7%	-	+		+	+	+		+			
Jana et al^20^	0%	85 %	+	+		+	+	+					
Bhutani et al^21^	72%	66%	-	-		+	+	-					
Sharma et al^22^	72%	66%	-	-		-		-					
Tekin et al^23^		67%	+	+		+		+		-			
Nassar et al^24^		86.0%	-	-	-	-	-	+		-			
Ameen et al^25^		48%	-	-	-	-	-	-		+			
Solanki et al^26^		58%		+			+	+		+			
Gao et al^27^		68.66%	-			+	+	-					
Sicking et al^28^		24.9%	-			+	-						+
Giaginis et al^29^		76.9%	-	-	-	↓+	-	-					↓+
Simonsson et al^30^		91%	+	-		↓+		-					-
Misron et al^31^		75%	-	-		↓+		-		-			
Serra et al^32^		66.9%	-	-			-	-					-
Aggarwal et al^33^		70%				-	-	-	-			-	

BC: breast cancer

A total of 17 studies demonstrated an important relationship between the phenotype of COX-2 and one or more of the histopathologic parameters of BC cases such as age, tumor size, histological type, tumor grade, advanced stage, lymph nodes metastasis, lymphovascular invasion, distant metastasis, surgical margins, and disease recurrence or shorter DFS.^17-21,23-27^ On the other hand, 3 studies found an inverse association with grade and DFS, and few other reports could not find such relationships.^22,28,30-33^

Here we detected an important relationship between increased COX-2 immunohistochemical expression and advance stage, metastases and lymphovascular invasion, which characterizes aggressive types of BC. In respect of the association with tumor stage, our outcomes are in line with the following earlier studies and contradict the following reports, which could not find such association.^19-21,24-29,32,33^ Only 3 studies examined the relationship among COX-2 immunoexpression and distant metastases, of which 2 studies found statistical significance and were in line with the current report, while only one study opposed these results.^17,18,33^ A total of 7 studies attempted to link the immunoexpression of COX-2 with lymphovascular invasion, the results of 4 studies supported our finding and showed significant association with COX-2 expression while the other 7 reports failed to do so.^18,19,23-26,31^

Regarding DFS, 6 studies used a log-rank comparison test to reveal substantial different survival distributions for several scores of COX-2 immunostainings, of which 3 studies showed that COX-2 immunohistochemical phenotype is allied with bad survival significantly.^17,18,28^ Our results support these 3 studies and contradicted the remaining 3, which one of them found an inverse association.^29,30,32^

### Study limitations

Although our findings are encouraging, our investigation and the other 17 reports have several limitations such as the sensitivity of utilized techniques, populations diversity, sample size variations, inconsistent scoring methods, and the semi-quantitative evaluation of staining. Still, multicentre research with a larger number of cases is positive and huge value for evaluating the clinical importance of COX-2 staining in the detection and prognoses of BC.

In conclusion, COX-2 is a valuable marker that could support BC diagnosis and prognosis. Its expression associated with several clinicopathological factors which distinguish aggressive subtypes of BC, such as advanced stage, distant metastases, lymphovascular invasion, and poor survival.
